# Comparison of Maximum Stretch Forces between Femtosecond Laser-Assisted Capsulotomy and Continuous Curvilinear Capsulorhexis

**DOI:** 10.1155/2017/3489373

**Published:** 2017-01-22

**Authors:** Mari Takagi, Takashi Kojima, Kei Ichikawa, Yoshiki Tanaka, Yukihito Kato, Rie Horai, Akeno Tamaoki, Kazuo Ichikawa

**Affiliations:** ^1^Department of Ophthalmology, Japan Community Healthcare Organization Chukyo Hospital, Nagoya, Japan; ^2^Department of Ophthalmology, Japanese Red Cross Gifu Hospital, Gifu, Japan; ^3^Chukyo Medical Co., Inc., Nagoya, Japan; ^4^Chukyo Eye Clinic, Nagoya, Japan; ^5^Shinshu University Interdisciplinary Graduate School of Science and Technology, Nagano, Japan

## Abstract

The current study reports comparing the postoperative mechanical properties of the anterior capsule between femtosecond laser capsulotomy (FLC) and continuous curvilinear capsulorhexis (CCC) of variable size and shape in porcine eyes. All CCCs were created using capsule forceps. Irregular or eccentric CCCs were also created to simulate real cataract surgery. For FLC, capsulotomies 5.3 mm in diameter were created using the LenSx® (Alcon) platform. Fresh porcine eyes were used in all experiments. The edges of the capsule openings were pulled at a constant speed using two L-shaped jigs. Stretch force and distance were recorded over time, and the maximum values in this regard were defined as those that were recorded when the capsule broke. There was no difference in maximum stretch force between CCC and FLC. There were no differences in circularity between FLC and same-sized CCC. However, same-sized CCC did show significantly higher maximum stretch forces than FLC. Teardrop-shaped CCC showed lower maximum stretch forces than same-sized CCC and FLC. Heart-shaped CCC showed lower maximum stretch forces than same-sized CCC. Conclusively, while capsule edge strength after CCC varied depending on size or irregularities, FLC had the advantage of stable maximum stretch forces.

## 1. Introduction

To achieve successful results in contemporary cataract surgery, precise correction of refractive error is essential, especially when toric or multifocal intraocular lenses (IOLs) are implanted. Furthermore, to calculate IOL power accurately, it is necessary to precisely predict the postoperative IOL position [1]; in this regard, previous literature has reported that IOL decentration or tilt can increase refractive error by inducing myopic shift or astigmatism [2, 3].

Many techniques have been used to create anterior capsule openings in cataract surgery, for example, continuous curvilinear capsulorhexis (CCC), radiofrequency diathermy, vitrectorhexis, and femtosecond laser-assisted capsulotomy (FLC). To prevent posterior capsule opacity (PCO) and IOL tilt, it is important that the IOL is completely covered by the capsular edge [4, 5]. Specifically, CCC is a major method of creating capsule openings in cataract surgery. However, recent studies have indicated that the femtosecond laser can create accurately sized, highly reproducible, and circular capsulotomies [6, 7].

During cataract surgery, intracapsular manipulation—such as what occurs during division of the nucleus and IOL implantation—stretches the edges of the capsule openings. In cases of zonular weakness, capsular stabilization devices also stretch the capsular edge.

The shape of CCCs varies depending on many factors. Previous reports have shown that the most common minor complications of cataract surgery performed by residents were irregular capsulorhexis and iris prolapse [8]. Generally, the effect of corneal refractive power allows surgeons to see a magnified image of the anterior capsule through a microscope. Indeed, one report showed that, due to this effect, keratometric power and axial length affected CCC size and position. Moreover, another investigation indicated that CCC tended to be eccentric in eyes with longer axial length [7]. However, the same author reported that FLC was not associated with these factors [7].

To the best of our knowledge, no studies have compared capsule edge strength between FLC and CCC of different sizes and irregularities. In this study, to evaluate stretch forces in real cataract surgery, we created CCC of different sizes, irregularities, and eccentricities; we then compared stretch forces between FLC and CCC.

## 2. Materials and Methods

We obtained fresh porcine eyes that had been enucleated less than 12 hours before the experiment; we then performed manual continuous curvilinear capsulorhexis or femtosecond laser-assisted capsulotomy in the following ways.

### 2.1. Manual Continuous Curvilinear Capsulorhexis

A single well-trained cataract surgeon performed all CCCs using capsule forceps. After creating a corneal incision using a 2.5 mm knife (BD Biosciences), the surgeon filled the anterior chamber using viscoelastic material. To maintain the capsule opening at the correct size, a ring caliper with a diameter of 5.3 mm (Ring Caliper Type 5®; MORCHER) was inserted into the anterior chamber and used as a guide to create the CCC.

**Figure 1:**
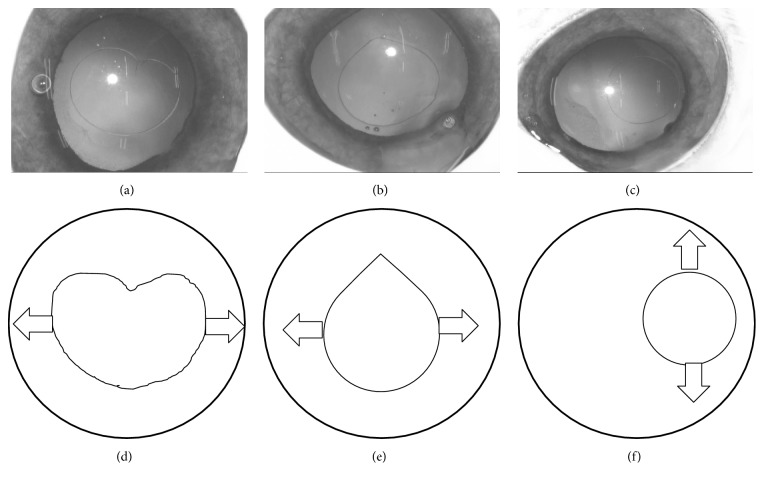
Representative shapes of irregular-shaped continuous curvilinear capsulorhexis (CCC). Three types of irregular CCC—heart-shaped (a), teardrop-shaped (b) and eccentric (c)—are shown. (d), (e), and (f) show the location of 2 jigs pulling capsule edges (arrows).

Having reviewed over 400 cataract surgeries that had been performed at the Department of Ophthalmology of the Japan Community Healthcare Organization Chukyo Hospital, we categorized irregular CCCs into three subgroups: teardrop-shaped CCC, heart-shaped CCC, and eccentric CCC. On this basis, we created irregular CCCs (teardrop-shaped: *n* = 12; heart-shaped: *n* = 11; eccentric: *n* = 10) using the same ring caliper to maintain the capsule opening size. Representative cases of irregular CCCs are shown in Figure 1.

### 2.2. Femtosecond Laser-Assisted Capsulotomy

All femtosecond laser-assisted capsulotomies were created using the LenSx (Alcon). In all cases, the Soft Fit® interface was applied to the eyes. After the LenSx had been docked onto the eyes, a 5.3 mm diameter capsulotomy was created under the following parameters: energy 6 *μ*J, spot separation 3 *μ*m, and layer separation 4 *μ*m—from 300 *μ*m above the lens to 350 *μ*m below the lens.

### 2.3. Image Analyses

After the FLC or CCC had been created, the ring caliper was inserted into the anterior chamber and placed onto the capsule openings under a surgical microscope. This procedure was recorded using a digital hard disk drive recorder; a still image was then obtained from the movie file. Using ImageJ software (1.47v, NIH), we measured the diameter, perimeter, and area of the capsule openings. On the basis of these values, we calculated the coefficient of variation (CV) of the capsule opening diameter, as well as the circularity of capsule opening, as described in previous reports [9]. In the eccentric CCC group, we measured the distance between the center of the CCC and the center of the pupil. For regular-shaped CCCs, the eyes were divided into three subgroups based on the achieved CCC diameter (small CCC subgroup: <4.8 mm; middle CCC subgroup: 5.3  ±  0.5 mm; large CCC subgroup: >5.8 mm).

### 2.4. Capsule Stretch Experiment

**Figure 2:**
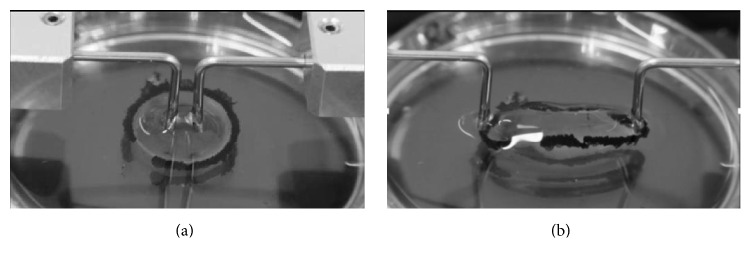
Images of the capsule stretch experiment. Two L-shaped jigs are inserted into the capsule opening (a); one side is pulled at constant speed (b).

After creating the FLC or CCC, we completely removed the crystalline lens material using a phacoemulsification device (WHITESTAR Signature; Abbott Medical Optics). Next, we injected viscoelastic material into the capsule and anterior chamber, and we cut the sclera near the equator to approach the crystalline lens from the posterior side. We released the zonule adhesions using surgical scissors and removed the crystalline lens capsule using a dispending spoon. The extracted whole capsule was then immersed in balanced salt solution. Two L-shaped jigs were created and inserted into the capsule openings; these were then pulled at constant speed (30 mm/min) using multipurpose material testing equipment (IMC-90F0; Imoto Machinery Co., Ltd.; Figure 2). We recorded stretch forces and distance over time, and the maximum values in this regard were defined as those that were recorded when the capsule broke. For the irregular CCCs, two jigs were placed and pulled in the specified position as shown in Figure 1 to avoid the direct force at the irregular part of CCC or equatorial part of lens capsule.

## 3. Statistical Analysis

The Mann–Whitney* U* test was used to compare stretch forces between two groups.

To compare values between three groups or more, the Kruskal–Wallis test was used. Dunn's multiple comparison test was performed as a post hoc test. To analyze the correlation between decentered distance and stretch forces in eyes with an eccentric CCC, we used Spearman's rank correlation coefficient. A *p* value less than 5% was considered statistically significant.

## 4. Results

In all porcine eyes in the FLC group, complete capsulotomies were created. The CCCs were also performed without any complications. When the crystalline lens was removed, none of the eyes developed anterior capsule tears or posterior capsule ruptures. The average CCC diameters were 4.27 ± 0.32 mm, 5.38 ± 0.22 mm, and 6.58 ± 0.25 mm in the small CCC, middle CCC, and large CCC subgroups, respectively.

**Figure 3:**
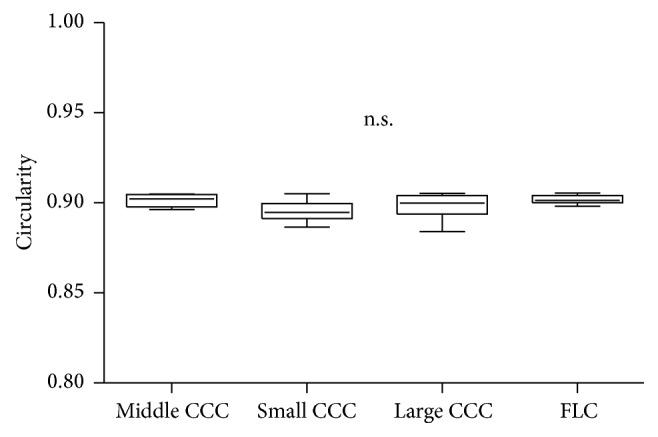
Comparison of capsule opening circularity among the FLC group and the small CCC, middle CCC, and large CCC subgroups. There were no significant differences among the groups. FLC: femtosecond laser capsulotomy; CCC: continuous curvilinear capsulorhexis.

The CV of diameter in the FLC group (0.098) was smaller than that in the CCC group (0.407). The CVs of diameter in the small CCC, middle CCC, and large CCC subgroups were 0.318 , 0.230, and 0.335, respectively. Circularity in the CCC and FLC groups is shown in Figure 3. There were no significant differences in circularity among the three CCC subgroups and FLC group (*p* = 0.0608).

**Figure 4:**
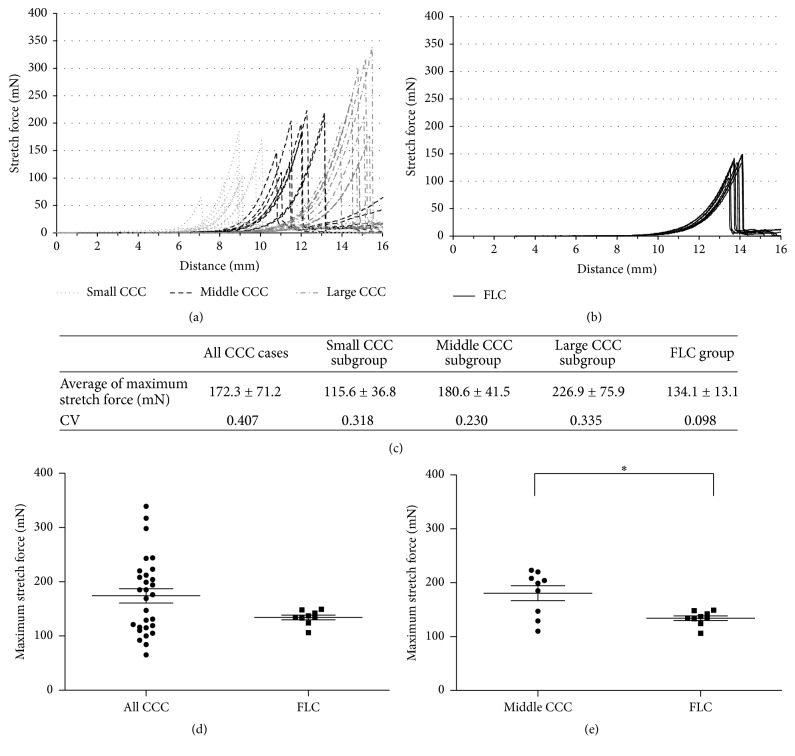
Results of the capsule stretch experiments. Association between stretch distance and stretch forces during the capsule stretch experiments in the CCC group (a) and FLC group (b) is shown. There were large variations in maximum stretch force and maximum stretch distance within the CCC group, while there was less variation in the FLC group in this regard. The average maximum stretch force and the coefficient of variation (CV) in each group are shown (c). There were no differences in mean stretch force between the CCC group and the FLC group (d). However, the middle CCC subgroup showed higher stretch forces than the FLC group (e). ^*∗*^*p* < 0.05.

Figure 4 shows the results of the capsule stretch experiment in the CCC and FLC groups. The large CCC subgroup showed higher maximum stretch forces. On the other hand, the FLC group showed less variation than all CCC subgroups in terms of maximum stretch force or maximum stretch distance. There was no significant difference in maximum stretch force between the CCC group (172.28 ± 71.2 mN) and the FLC group (134.11 ± 13.13 mN). However, the maximum stretch force in the middle CCC subgroup (180.56 ± 41.51 mN), in which the CCC diameter was similar to that of the FLC group, was significantly higher than that in the FLC group (134.11 ± 13.13 mN) (*p* = 0.038).

**Figure 5:**
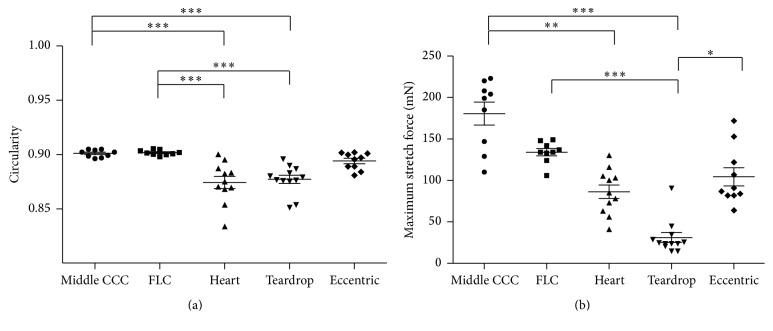
Comparison of circularity (a) and stretch force (b) among the CCC subgroups and the FLC group. The heart- and teardrop-shaped CCC subgroups showed significantly lower circularity than the middle CCC subgroup and FLC group. The maximum stretch force in the teardrop-shaped CCC subgroup was significantly lower than those in the middle CCC subgroup, FLC group, and eccentric CCC subgroup. The maximum stretch force in the heart-shaped CCC subgroup was significant lower than that in the middle CCC subgroup. ^*∗*^*p* < 0.05, ^*∗∗*^*p* < 0.001, and ^*∗∗∗*^*p* < 0.0001.

Circularity in the heart-shaped CCC subgroup was significantly lower than that in the middle CCC subgroup and FLC group (Figure 5, *p* < 0.0001 in both cases). Similarly, circularity in the teardrop-shaped CCC subgroup was significantly lower than in the CCC middle subgroup and FLC group (*p* < 0.0001 in both cases). Next, we compared the maximum stretch forces among the irregular CCC subgroup, the middle CCC subgroup, and the FLC group. The mean maximum stretch force in the heart-shaped CCC subgroup was significantly lower than that in the middle CCC subgroup (*p* < 0.001). The mean maximum stretch force in the teardrop-shaped CCC subgroup was significantly lower than those in the middle CCC subgroup, the FLC group, and the eccentric CCC subgroup (Figure 5; *p* < 0.0001, *p* < 0.0001, and *p* < 0.05, resp.).

**Figure 6:**
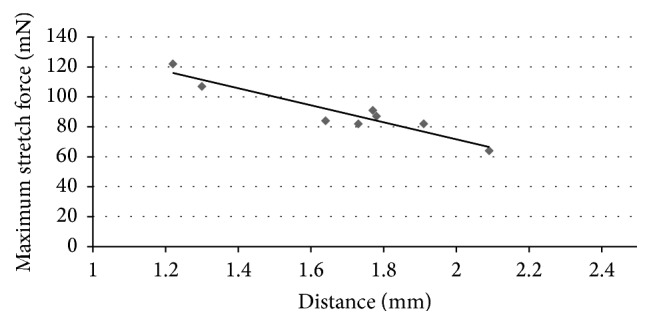
The correlation between eccentric distance and maximum stretch force in the eccentric CCC subgroup. There was a significant negative correlation between eccentric distance and maximum stretch force (*r* = −0.7785, *p* = 0.0279).

After two outliers were excluded, we found a significant negative correlation between the decentered distance and the maximum stretch force (Figure 6; *r* = −0.7785; *p* = 0.0279).

## 5. Discussion

To achieve minimum postoperative refractive error after cataract surgery, it is essential that surgeons accurately predict the effective lens position and axial length [1]. Similarly, to ensure that the IOL's position is accurately predicted, it should be properly implanted in the capsule. The edges of the capsule openings are often stretched during cataract surgery. Resultant capsule tearing causes IOL decentration, tilt, and PCO, which can lead to visual impairment [2, 3, 10, 11].

In previous studies, there have been discrepancies between FLC and CCC in relation to the stretching forces [12–14]. Some investigations have shown that CCC confers higher stretch forces than FLC, suggesting that CCC creates histologically smoother capsular edges than FLC [15–17]. Conversely, several other studies have found that FLC confers higher stretch forces than CCC, proposing that the round shape of the capsulotomy in FLC contributes to a more uniform stress distribution than in CCC [6, 13, 18].

In the current study, the large CCC subgroup showed higher maximum stretch force than did the small CCC subgroup. This result was consistent with that of a previous report [14]. We speculated two reasons for this result. First, the circumference of the capsule opening may itself affect the maximum stretch force. Second, the fact that the midperipheral part of the porcine anterior lens capsule is thicker than the central part may also have an influence [19]. In the present study, the large CCC subgroup showed higher variability in stretch force than did the middle CCC subgroup, perhaps because the peripheral lens capsule varied more in thickness than did the central part of lens capsule. When we included CCC of variable sizes, there was no difference in maximum stretch forces between FLC and CCC. Average value of maximum stretch forces in CCC and FLC group was 172.3 mN and 134.1 mN, respectively, which seemed to indicate a large difference. However, no difference between the two groups was observed probably because the CCC group showed greater variability in maximum stretch forces than did the FLC group. However, when we compared the maximum stretch forces between the FLC group and the same-sized CCC subgroup, the CCC subgroup yielded greater values than did the FLC group. On the basis of these results, we must conclude that the advantage of FLC is consistency in capsule edge strength. Nonetheless, CCC did confer greater capsule edge strength than FLC.

The principle of femtosecond laser incision is called photodisruption, in which plasma creates cavitation bubbles that separate the tissue. Histological evaluation of capsular edges using scanning electron microscopy revealed that photodisruption had caused irregularities such as tags and notches in the capsular edges [12, 15–17, 20, 21]. We would suggest that these features of femtosecond laser incision affect the maximum stretch forces at the anterior lens capsule edge.

We also found that the maximum stretch forces in the teardrop-shaped CCC subgroup were lower than those in the regular-shaped CCC subgroup and same-sized FLC group, suggesting that circularity and completeness of the CCC are essential for achieving higher strength at the capsular edge. In particular, the teardrop-shaped CCC subgroup also showed lower maximum stretch forces than the eccentric CCC subgroup. We did not find any significant difference between the teardrop-shaped and heart-shaped CCC subgroups; however, the teardrop-shaped CCC subgroup did seem to have lower stretch forces than the other subgroups. To explain this difference, we posit that the capsule edge after teardrop-shaped CCC tears easily when stretch forces are applied, perhaps because it is weak at the point of the outward notch. These results also suggested that continuity of the capsulotomy or CCC is more important than the microstructure of the capsulotomy edges and thickness of the capsule.

In the eccentric CCC subgroup, after excluding two outliers, we found a significant negative correlation between decentered distance and stretch forces. By way of explanation for this correlation, we suspect that when the CCC was created more peripherally, it included a thinner part of the lens capsule. The two outlier CCCs showed large decentered distance (1.99 mm and 2.13 mm, resp.), as well as high maximum stretch force (172 mN and 153 mN, resp.). Based on our analyses of the video that was recorded during the stretch experiment, we believe that the equator of lens capsule may have interfered with the stretch experiment.

Initially, we expected that the eccentric CCC subgroup would have lower capsule edge strength than the centric CCC subgroup. However, we could not find any significant differences in this regard, perhaps because the decentered distance varied in the eccentric CCC subgroup. If we had created eccentric CCCs with a predetermined large decentered distance, the maximum stretch force in the eccentric CCC subgroup may have been lower than those in the well-centered CCC subgroup.

There were several limitations in the current study. Firstly, we used a single femtosecond laser platform for the experiment. Previous research has shown that the interface between the femtosecond laser machine and the patient can affect histological differences at the capsule edges [20]. Therefore, a different femtosecond laser may have yielded different stretch force results. In the future, we need to further evaluate stretch forces in the anterior lens capsule using different femtosecond laser machines with different patient interfaces.

Secondly, we used a consistent femtosecond laser setting for the current experiment. Several reports showed that FLCs with high energy lead to an irregular cutting edge and that they decrease maximum stretch forces [21, 22]. Furthermore, future studies should evaluate the effect of laser energy or spot/layer separation settings on capsule stretch forces. Thirdly, we performed the lens capsule stretch experiment using porcine eyes, whereas Parel et al. reported that the maximum stretch force in the large CCC subgroup was larger than that of the small CCC subgroup of human cadaver eyes [23]. This corroborates the current study and shows a similarity between the porcine and human crystalline lens capsules. However, according to the previous literature, the lens capsule in porcine eyes is thicker than that in human eyes, and the elasticity of the porcine lens capsule is similar to that in human infants [24, 25]. Moreover, the human lens capsule becomes thicker with aging [25, 26], but maximum stretch force is negatively correlated with age [26]. Since the current study used porcine eyes, it is not clear how relevant the results are to real cataract surgery. Further experiments using human eyes and taking account of donor age are needed in the future.

The shape of the capsule opening often becomes irregular, too small, or too large, especially in patients with small pupils, shallow anterior chamber depth, zonular weakness, white cataract, mature cataract, pediatric cataract, or poor visibility by low red reflex. In the current study, irregular-shaped CCCs showed lower capsule strength than well-centered and round-shaped CCCs; therefore, irregular-shaped CCCs may increase the risk of capsule tear, which can cause IOL decentration and tilt. We believe that one advantage of FLC is that it involves a higher percentage of capsule overlap, ensuring that the IOL has less tilt and better centration than in CCC, as previously described [2, 9, 27, 28].

Several studies have shown that FLC has a high success rate and that its strength and size are stable [29]. In one study, the size of the capsule openings was related to anterior chamber depth after cataract surgery [30], and a stable-sized capsulotomy may also allow IOL power to be more accurately calculated.

In conclusion, the current study revealed that FLC confers stable capsule edge strength and that the technique has advantages over teardrop-shaped CCC.
